# Supplementary material for: Intestinal Microbiome Associated with Efficacy of Atezolizumab and Bevacizumab Therapy for Hepatocellular Carcinoma
Source: Cancers (Basel). 2024 Apr 26;16(9):1675. doi: 10.3390/cancers16091675 (PMC11083184; doi:10.3390/cancers16091675)
Supplement: Supplementary file 1 [file cancers-16-01675-s001.zip › Supplemental figure.pdf]

Supplementary Figure S1

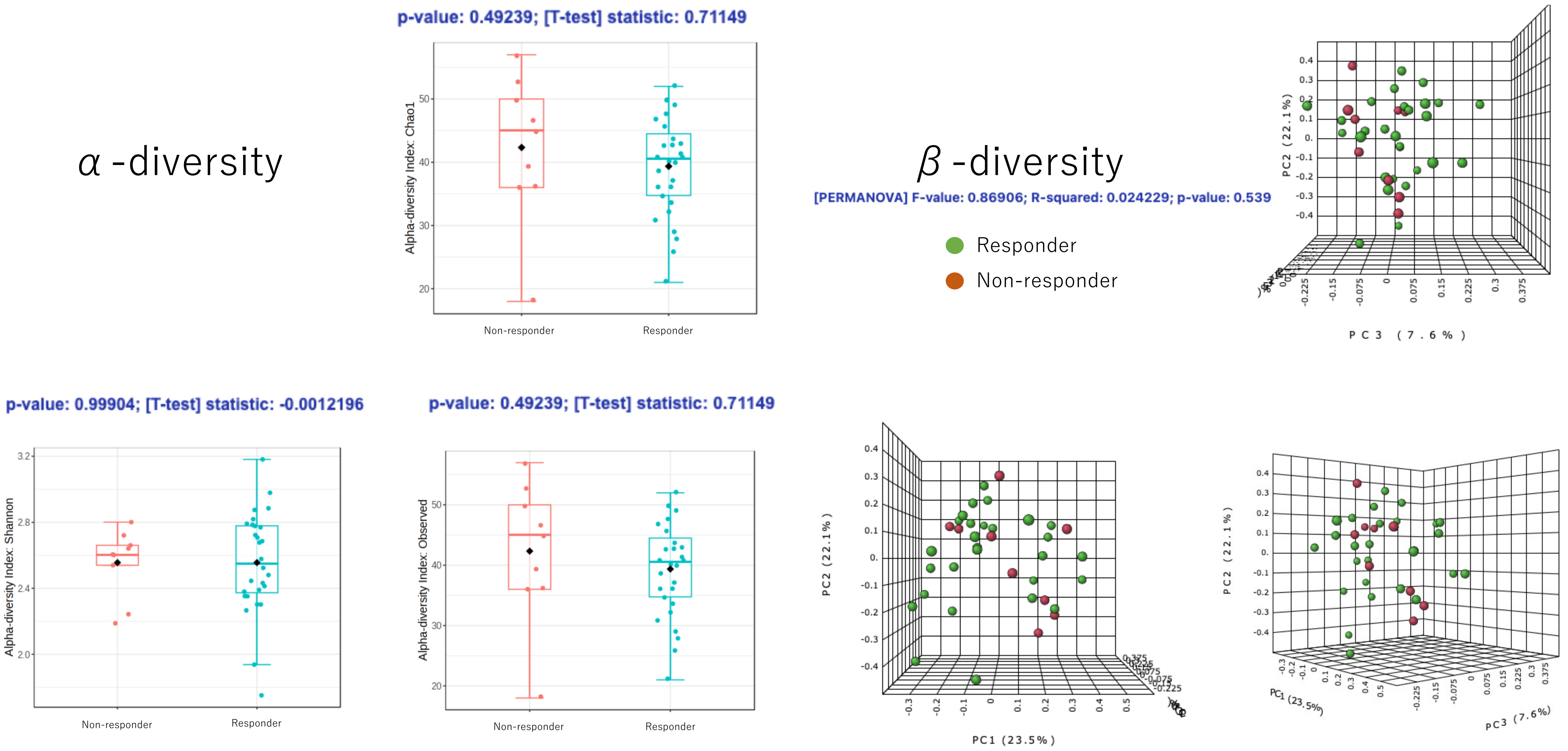

Supplementary Figure S2

Bacteroides stercoris

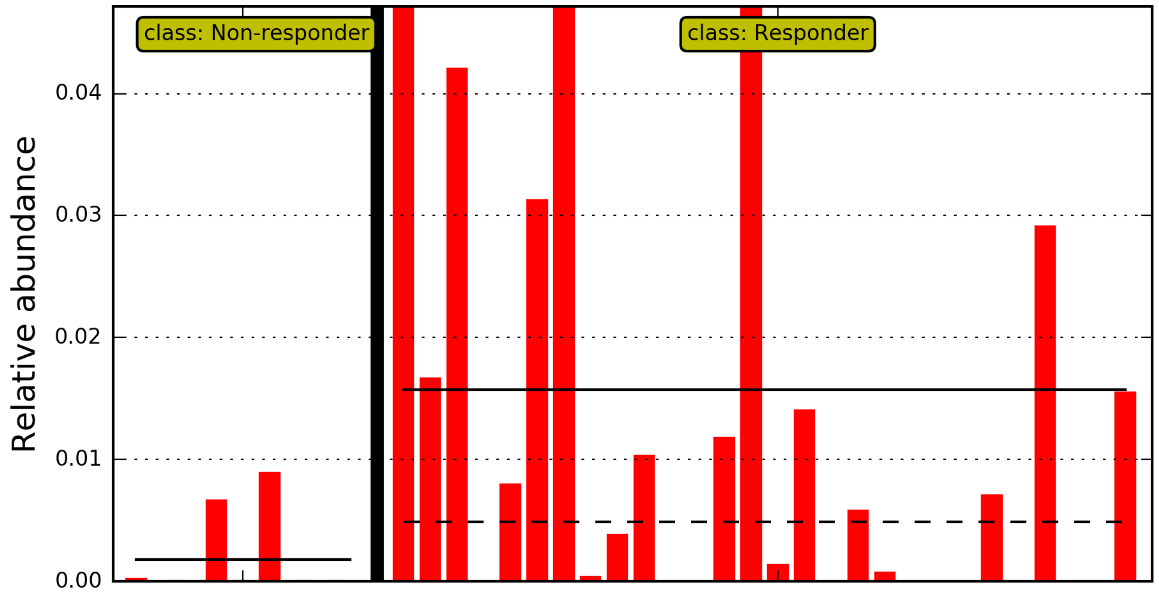

Parabacteroides merdae

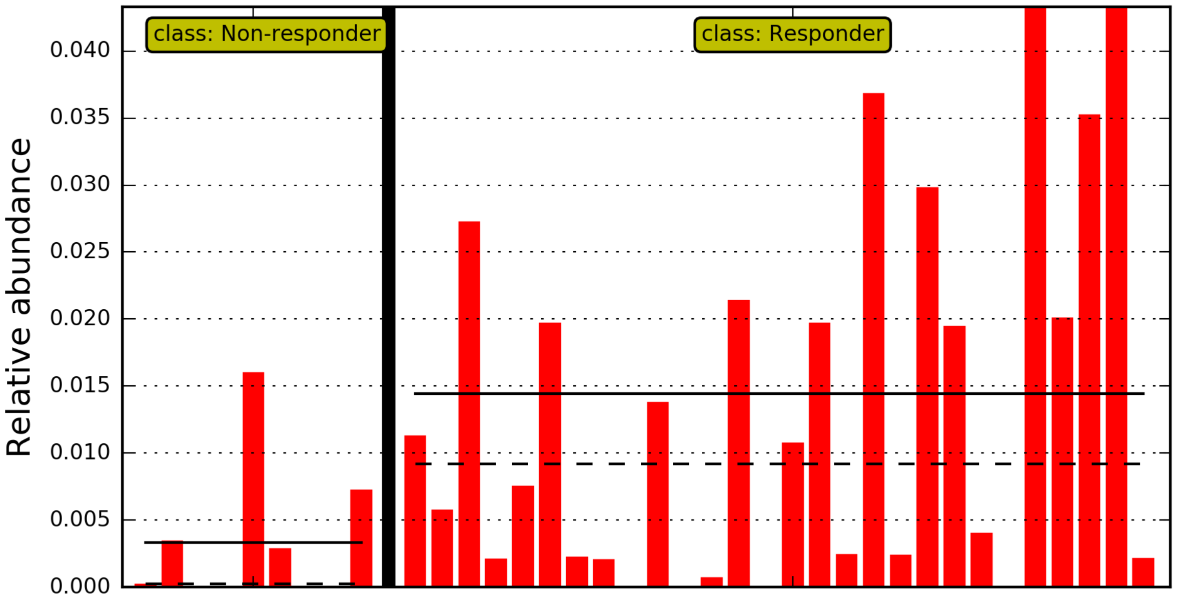

Supplementary Figure S3

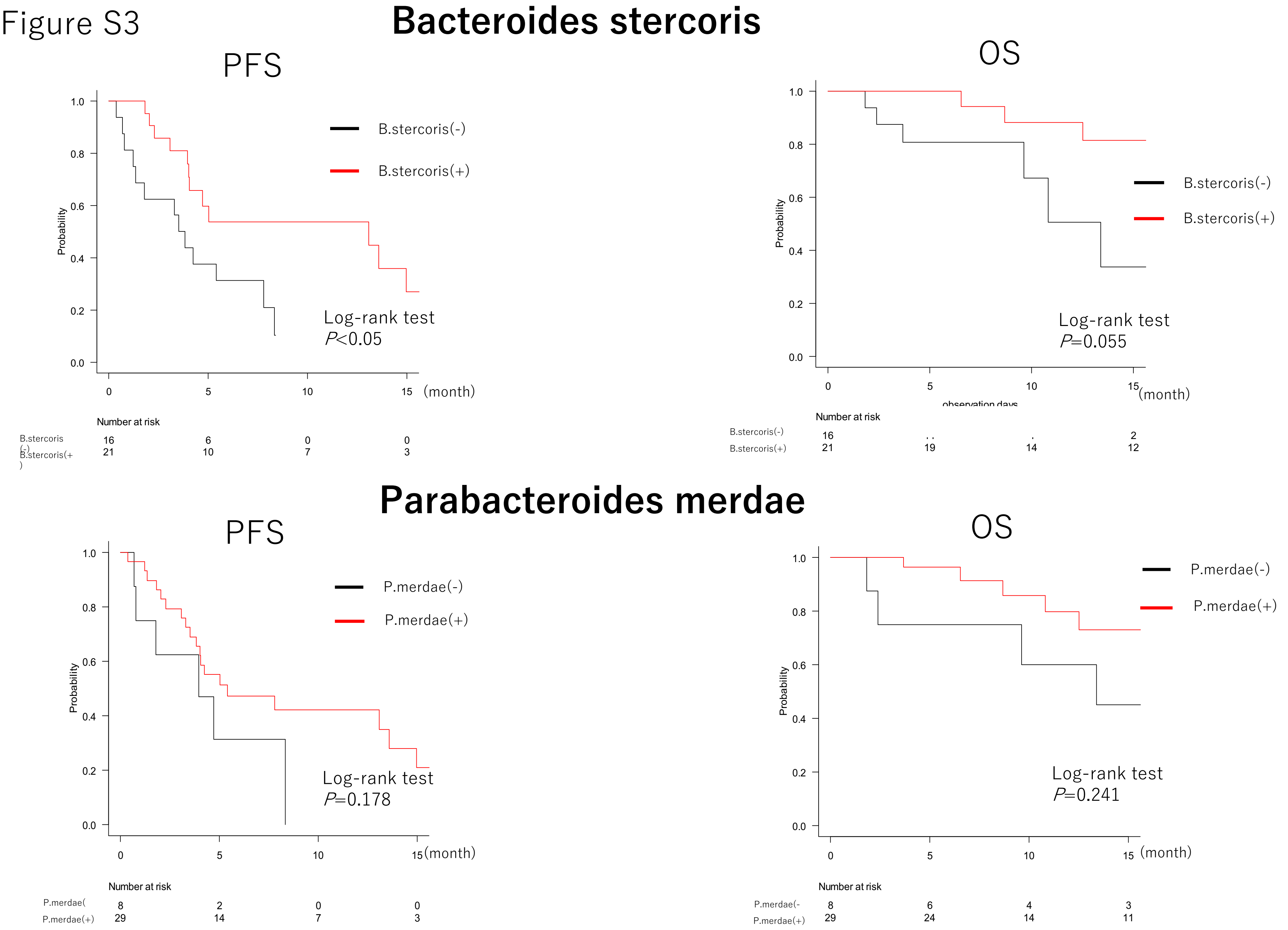

Supplementary Figure S4

PFS

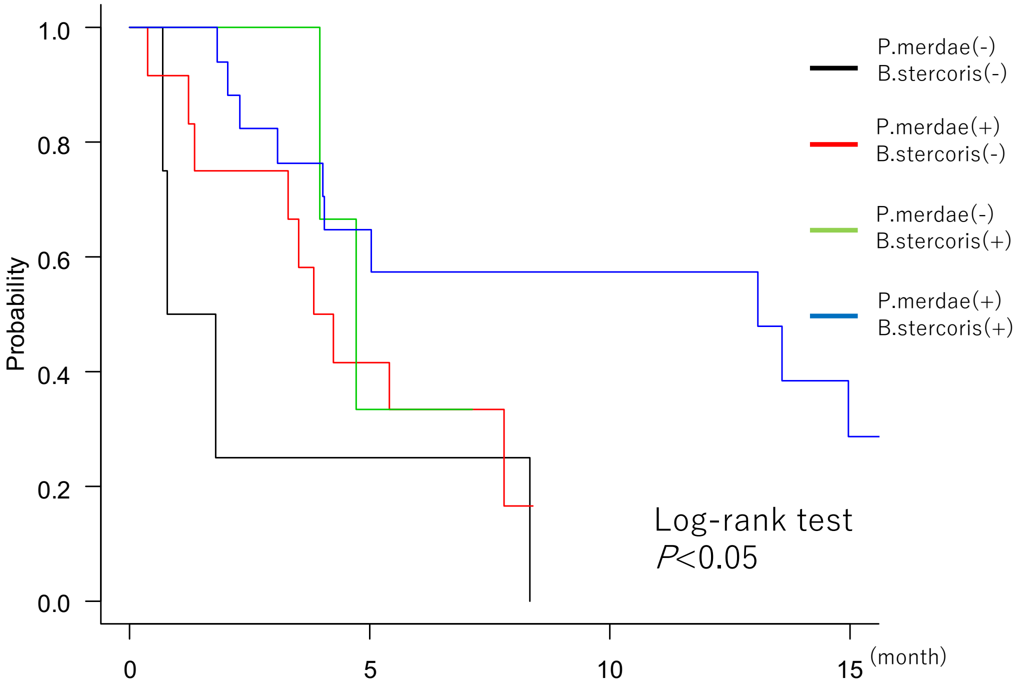

Number at risk

	0	5	10	15
P.merdae(-) B.stercoris(-)	4	1	0	0
P.merdae(+) B.stercoris(-)	12	5	0	0
P.merdae(-) B.stercoris(+)	4	1	0	0
P.merdae(+) B.stercoris(+)	17	9	7	3

OS

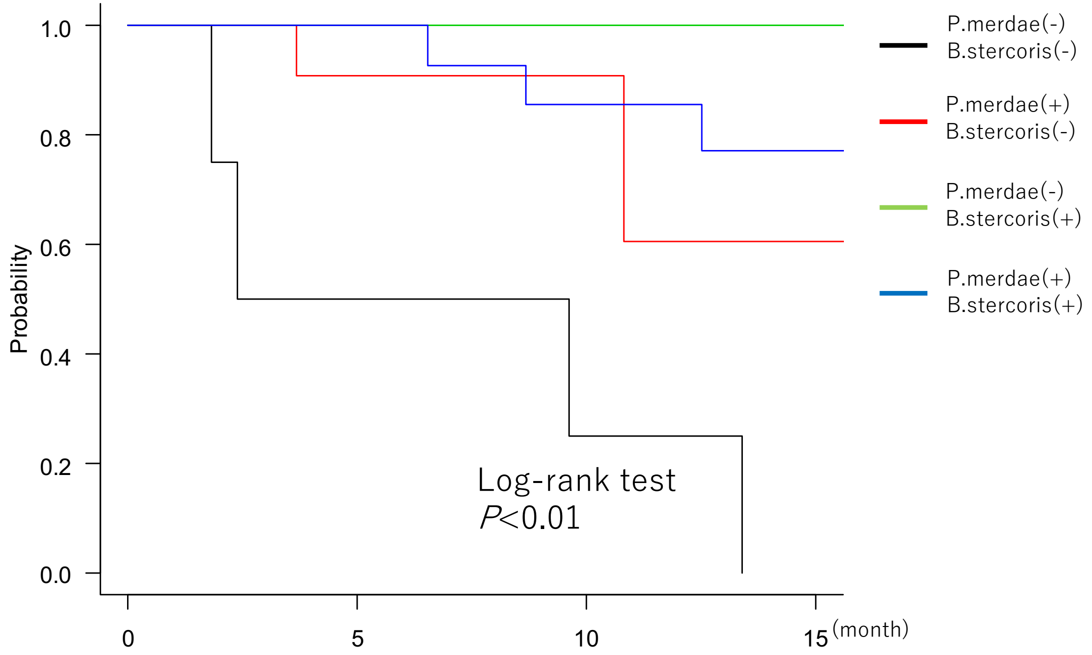

Number at risk

	0	5	10	15
P.merdae(-) B.stercoris(-)	4	2	1	0
P.merdae(+) B.stercoris(-)	12	9	3	2
P.merdae(-) B.stercoris(+)	4	4	3	3
P.merdae(+) B.stercoris(+)	17	15	11	9
