# Supplementary material for: Intestinal Microbiome Associated with Efficacy of Atezolizumab and Bevacizumab Therapy for Hepatocellular Carcinoma
Source: Cancers (Basel). 2024 Apr 26;16(9):1675. doi: 10.3390/cancers16091675 (PMC11083184; doi:10.3390/cancers16091675)
Supplement: Supplementary file 1 [file cancers-16-01675-s001.zip › Supplementary Table.pdf]

Supplementary Table S1

	P.merda(-) B.stercoris(-) n=4	P.merdae(+) B.stercoris(-) n=12	P.merdae(-) B.stercoris(+) n=4	P.merdae(+) B.stercoris(+) n=17	*P*-value
Age †	83(79-85)	74(69-79)	67(60-73)	74(60-79)	0.134
Gender (male/female)	2/2	9/3	3/1	14/3	0.604
Body mass index*	21.7(20.1-23.3)	22.3(20.5-24.3)	26.8(25.0-27.3)	24.6(22.5-28.8)	0.122
Etiology (HBV/HCV/Alcohol/NBNC)	3/0/1/0	2/4/5/1	1/2/0/1	3/4/5/5	0.470
BCLC stage (A/B/C)	0/1/3	0/4/8	0/0/4	3/4/10	0.451
Child-Pugh grade (A/B)	3/1	12/0	4/0	15/2	0.355
Child-Pugh score (5/6/7)	1/2/1	11/1/0	2/2/0	11/4/2	0.190
Treatment history (1/2/3/4)	3/0/0/1	10/2/0/0	3/0/0/1	14/3/0/0	0.192
AST (IU/L) †	134(88-174)	35(22-43)	23(17-28)	38(27-47)	0.069
ALT (IU/L) †	52(23-107)	25(15-31)	20(17-21)	30(27-38)	0.040
γ-GTP (IU/L) †	266(147-447)	44(33-94)	60(51-65)	102(70-150)	0.181
Total bilirubin (mg/dL) †	0.9(0.7-1.0)	0.7(0.6-1.0)	0.7(0.6-1.1)	0.8(0.7-1.2)	0.829
Albumin (g/dL) †	3.0(2.8-3.3)	3.8(3.5-4.1)	3.8(3.6-4.0)	3.7(3.6-4.0)	0.228
HbA1c (%) †	6.2(5.9-6.8)	6.1(5.7-6.5)	5.7(5.6-6.4)	6.3(6.1-6.8)	0.772
AFP (ng/mL) †	4336(430-36901)	670(5-981)	190(10-399)	7(5-13)	0.046
PPI (yes/no)	2/2	6/6	1/3	14/3	0.098
Antibiotics(yes/no)	0/4	2/10	1/3	1/16	0.537
C-reactive protein(mg/dL) †	1.27(0.82-1.47))	0.14(0.06-0.36)	0.11(0.07-0.17)	0.29(0.14-0.60)	0.116
NLR †	3.7(3.2-4.1)	1.7(1.3-2.9)	2.5(2.1-2.9)	1.8(1.6-2.8)	0.294

BCLC, the Barcelona Clinic Liver Cancer Classification ; AST, aspartate aminotransferase; ALT, alanine aminotransferase; γ-GTP, γ-glutamyl transpeptidase; AFP, α-fetprotein; PPI, protom pump inhibitor; NLR, Neutrophil-Lymphocyte Ratio

† Values are expressed as median (interquartile range).
